# Development of a transgenic mouse model of hepatocellular carcinoma with a liver fibrosis background

**DOI:** 10.1186/s12876-016-0423-6

**Published:** 2016-01-29

**Authors:** Sook In Chung, Hyuk Moon, Dae Yeong Kim, Kyung Joo Cho, Hye-Lim Ju, Do Young Kim, Sang Hoon Ahn, Kwang-Hyub Han, Simon Weonsang Ro

**Affiliations:** Institute of Gastroenterology, Yonsei University College of Medicine, Seoul, 120-752 South Korea; Brain Korea 21 Project for Medical Science College of Medicine, Yonsei University, Seoul, 120-752 South Korea; Department of Internal Medicine, Yonsei University College of Medicine, Seoul, 120-752 South Korea; Room 407, ABMRC, Severance Hospital, Yonsei University College of Medicine, Yonsei-ro 50-1, Seoul, 120-752 South Korea

**Keywords:** Transgenic mouse, Hepatocellular carcinoma, Fibrosis, Hydrodynamic transfection, Liver injury

## Abstract

**Background:**

Liver fibrosis and its end-stage disease, cirrhosis, are major risk factors for hepatocellular carcinoma (HCC) and present in 80 to 90 % of patients with HCC. Current genetically engineered mouse models for HCC, however, generally do not feature liver fibrosis, which is a critical discrepancy between human HCC and murine models thereof. In this study, we developed a simple transgenic mouse model of HCC within the context of a fibrotic liver.

**Methods:**

Employing hydrodynamic transfection (HT), coupled with the *Sleeping Beauty* (SB) transposon system, liver was stably transfected with transposons expressing cMyc and a short hairpin RNA down-regulating p53 (shp53). A chronic liver injury model, induced by hepatotoxic carbon tetrachloride (CCl_4_), was applied to the transgenic mice, allowing cells expressing cMyc plus shp53 to become malignant in the background of liver fibrosis.

**Results:**

Livers harvested about 3 months after HT had excessive collagen deposition and activated hepatic stellate cells surrounding the tumors. Hepatocarcinogenesis was significantly accelerated in the fibrotic livers compared to those of the control, significantly decreasing the life span of the mice. The tumor incidence and average number of tumors per mouse were significantly higher in the group treated with CCl_4_ compared to the vehicle-treated control mice, following HT (*p* < 0.01).

**Conclusions:**

Considering the simplicity and efficiency in generating HCC for fibrotic livers, the transgenic HCC model has the potential to be effectively used in preclinical testing of HCC anticancer therapy and in studies of hepatocarcinogenesis in fibrotic livers.

**Electronic supplementary material:**

The online version of this article (doi:10.1186/s12876-016-0423-6) contains supplementary material, which is available to authorized users.

## Background

Hepatocellular carcinoma (HCC) is one of the most prevalent and lethal cancers worldwide, ranking third among all cancer-related mortalities and accounting for 500,000 deaths annually [1, 2]. Most patients with HCC have a long history of chronic liver disease caused by diverse factors including hepatitis B and C viral infection, alcohol abuse, diabetes, and obesity [3–6]. Persistent injury to the liver from such factors leads to fibrotic scars in the tissue, characterized by an excessive accumulation of collagen fibers in the space of Disse [7, 8]. Liver fibrosis and its end-stage disease, cirrhosis, are highly associated with HCC. Fibrosis and cirrhosis are present in 80 to 90 % of patients with hepatocellular carcinoma and the 5-year cumulative risk for the development of HCC in patients with cirrhosis is between 5 and 30 % [2, 9].

Genetically engineered mouse (GEM) models for HCC have been generated for activated oncogenic signaling pathways or inactivated tumor-suppressing pathways, making significant contributions to our understanding of the genetic mechanism underlying the pathogenesis [10, 11]. Development of a GEM model usually involves expensive and time-consuming processes, such as genetic manipulation of target cells, subsequent implantation, and breeding of the animal. Furthermore, current GEM models for HCC generally do not feature liver fibrosis, calling into question whether the models can reliably recapitulate human HCC [10, 12]. Given the high association of chronic liver injury and fibrosis with the development of human HCC, a novel animal model is needed in which HCC is induced within the microenvironment of hepatic injury and fibrosis.

A very elegant and simple method was recently developed for liver-specific transgenesis in which the hydrodynamics-based transfection (HT) method was coupled with the *Sleeping Beauty* (SB) transposase system [13]. This simple liver-specific transgenic approach allowed generation of various HCC transgenic models with reduced time and resources [14]. In this study we developed a transgenic model for HCC via HT of transposons expressing cMyc and short hairpin RNA down-regulating *p*53 (shp53). To induce HCC within the context of hepatic injury and fibrosis, mice transfected with cMyc plus shp53 were repeatedly treated with carbon tetrachloride (CCl_4_), a hepatotoxic chemical that induces chronic liver damage [15, 16]. Using the mouse model, the effect of CCl_4_ treatment and liver fibrosis on hepatocarcinogenesis was investigated.

## Methods

### Animals

All experiments involving live mice were performed according to the Guidelines and Regulations for the Care and Use of Laboratory Animals in AAALAC-accredited facilities, and were approved by the Animal Policy and Welfare Committee of the Yonsei University College of Medicine (Permit number: 2014–0261). The mice were 5–to 6-week-old C57BL/6 males purchased from the Orientbio (Seongnam, Korea).

### Plasmids and hydrodynamic transfection

The plasmid pT2/GFP harboring a transposon encoding the enhanced green fluorescent protein (GFP) was described previously [17]. The cDNA encoding murine cMyc was PCR amplified from pCX-cMyc, a gift from Dr. Shinya Yamanaka (Addgene plasmid # 19772). The amplified cDNA replaced the GFP cDNA in pT2/GFP, generating pT2/cMyc. The pPGK-SB13 plasmid, encoding SB transposase under the control of the phosphoglycerate kinase (PGK) promoter, was a gift from Dr. John Ohlfest. The transposon plasmid, pT2/shp53/GFP4, which encodes a short hairpin RNA against the tumor suppressor p53, with GFP as a reporter, was a gift from Dr. John Ohlfest and is referred to as pT2/shp53 [18]. For hydrodynamic injection, 14 μg of pT2/cMyc and 14 μg of pT2/shp53 (or pT2/GFP as a control) were mixed with 9 μg of pPGK-SB13 and then suspended in 2 ml of Lactated Ringer solution. The DNA solution was injected into the lateral tail veins of 6-week-old mice (0.1 ml/g body weight) in less than 7 s.

### Carbon tetrachloride (CCl_4_) treatment

CCl_4_ was administered to mice twice weekly at a dose of 1 ml/kg body weight [16]. Mice were monitored regularly following administration of the CCl_4,_ and treated and sacrificed according to institutional guidelines.

### Liver harvest and tissue processing

Mice were deeply anesthetized by intraperitoneal injection of zoletil (30 mg/kg) and xylazine (10 mg/kg). Livers were harvested after a midline laparotomy incision and then carefully inspected for tumor nodules. Extracted livers were then immersed in 10 % neutral-buffered formalin. Fixed liver specimens were embedded in paraffin blocks.

### H&E staining and histopathological examination

Liver specimens embedded in paraffin blocks were sectioned into 4-μm slices, which were stained with hematoxylin and eosin (H&E) and picro-sirius red following standard protocols. Liver lesions were assessed as described by Frith et al. [19]. Slides were analyzed and photographed using a microscope (Eclipse Ti; Nikon, Tokyo, Japan) equipped with a digital camera.

### Western blotting

Liver tissues were homogenized and digested in 1× RIPA buffer containing phosphatase inhibitor cocktail solution (GenDEPOT, Barker, TX, USA). Western blot experiments were performed following the standard protocol. The following primary antibodies were used: anti-c-Myc (ab32072; Abcam, Cambridge, UK), anti-p53 (sc-6243; Santa Cruz Biotechnology, Santa Cruz, CA, USA), and anti-GAPDH (#2118; Cell Signaling Technology, Danvers, MA, USA). Anti-rabbit IgG–HRP (Sigma-Aldrich, St. Louis, MO, USA) was used as the secondary antibody. Bands were detected using the enhanced chemiluminescence (ECL) Western blot detection system (Amersham Pharmacia Biotech, Piscataway, NJ, USA).

### Immunohistochemistry

Paraffin sections were deparaffinized in xylene and rehydrated through a gradual decrease in concentration of ethanol. The antigen epitopes were then unmasked using sodium citrate buffer (pH 6.0). Subsequently, the sections were incubated overnight at 4 °C with the following primary antibodies: anti-α-smooth muscle actin (ab5694; Abcam), anti-c-Myc (ab32072; Abcam) and anti-GFP (#2555; Cell Signaling Technology). After primary incubation, sections were incubated with the appropriate biotinylated secondary antibodies, followed by treatment with freshly prepared DAB substrates (Vector Laboratories, Burlingame, CA, USA). Sections were lightly counter-stained with hematoxylin and mounted.

### Mouse survival and statistical analysis

Mice were monitored daily for illness symptoms. Kaplan–Meier survival data were analyzed using a log-rank test. Statistical analyses were conducted using an unpaired parametric Student’s *t*-test or Fisher’s exact test, as appropriate. A value of *p* < 0.01 was taken to indicate statistical significance.

## Results

### Mice expressing cMyc plus shp53 develop well-differentiated HCC

Transgenic mice were developed expressing cMyc and short hairpin RNA down-regulating *p53* (shp53) in the liver via hydrodynamics-based transfection. Inactivation of P53 has been frequently observed across the diversity of etiologic factors in human HCC [20, 21]. In particular, allelic deletions or mutations in *P53* have been frequently detected in hepatocellular carcinoma after HBV or HCV infection [22]. The Myc protein is a transcription factor that promotes cell proliferation and growth, and is overexpressed in up to 70 % of viral and alcohol-related HCC [23–25]. Thus, overexpression of cMyc and downregulation of *p53* in the model are genetic characteristics relevant to human hepatocarcinogenesis.

**Fig. 1:**
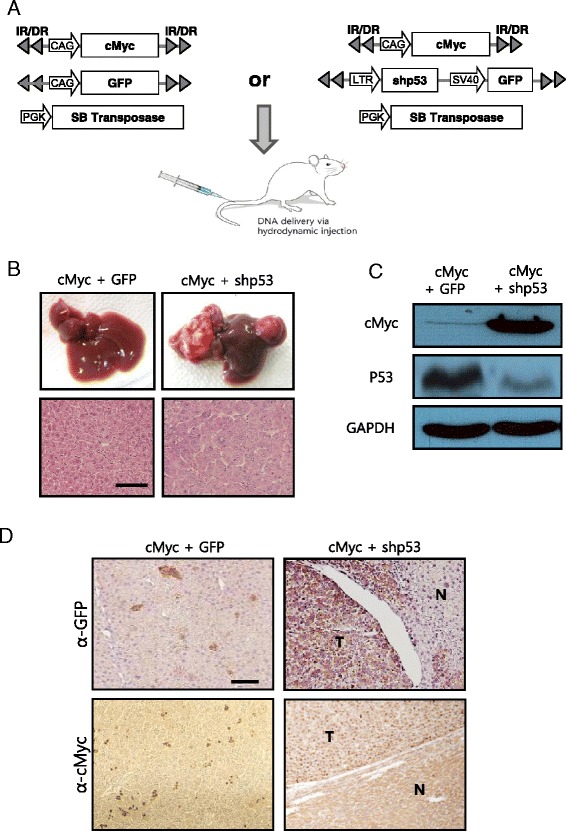
Expression of cMyc plus shp53 in the liver induces well-differentiated HCC. **a** Schematic illustration of the experimental procedure to generate transgenic livers expressing cMyc plus GFP (control), and cMyc plus shp53. Hydrodynamic transfection was performed using a mixture of indicated plasmids. **b** Gross morphology (upper panels) of livers harvested from mice of each group at 7 months post hydrodynamic injection. Images of H&E staining in liver sections are shown below. Well-differentiated HCCs developed in cMyc plus shp53 mice, while the control mice did not develop tumors. Scale bar, 100 μm. **c** Protein expression levels of cMyc and p53 in liver of cMyc plus GFP mice (control) and liver tumor of cMyc plus shp53 mice. **d** Images of IHC staining for GFP and cMyc in liver sections of indicated groups. “T” denotes tumor and “N” denotes non-tumorous liver parenchyma tissue. Scale bar, 100 μm

Transposons encoding cMyc plus shp53 (or GFP, as a control) were mixed with plasmids expressing the SB transposase and then hydrodynamically delivered to the liver (Fig. 1a). Livers were harvested at 7 months post-hydrodynamic injection (PHI). About 43.5 % (10 of 23) of the mice developed liver tumors in the cMyc plus shp53 group, while no mice in the cMyc plus GFP group (*n* = 10) had hyperplastic nodules in their livers (Fig. 1b). Liver tumors of cMyc plus shp53 mice showed overexpression of cMyc and down-regulation of *p53*, indicating that they originated from cells transfected with the oncogene-encoding transposons (Fig. 1c). Overexpression of cMyc and shp53 in tumors was confirmed via IHC staining for cMyc and GFP, respectively (Fig. 1d). IHC staining in liver sections from the cMyc plus GFP mice revealed scattered GFP-positive and cMyc-positive clusters consisting of 1–3 hepatocytes (Fig. 1d), suggesting that overexpression of cMyc alone was insufficient to induce tumor in the liver. Consistent with previous reports, no nodules were found in livers of the 10 control mice expressing shp53 alone, when analyzed at 7 months PHI (data not shown) [17]. Thus, the data indicate that oncogenic collaboration between cMyc overexpression and p53 down-regulation is required to induce tumors in the liver. Histopathological examination revealed that the tumors from the cMyc plus shp53 group exhibited typical features of highly differentiated hepatocellular carcinomas, with broadened trabeculae (Fig. 1b).

### Combining the transgenic HCC model with a chronic liver injury model

**Fig. 2:**
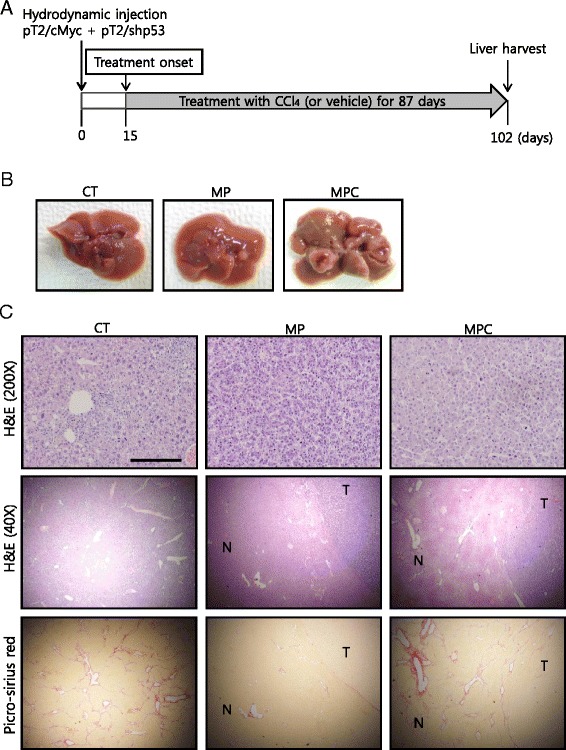
Application of a chronic liver injury model to the cMyc plus shp53 mice induces HCC in a fibrotic liver background. **a** Diagram of experimental procedures. Starting at 15 d post hydrodynamic transfection, mice were treated with CCl_4_ or the vehicle twice per week for about 12 weeks. **b** Gross images of representative livers of CT, MP, and MPC mice that were harvested at 102 d post hydrodynamic transfection. **c** H&E (200× and 40×) and picro-sirius red staining in liver sections of indicated groups. Tumors from both MP and MPC show similar well-differentiated HCC phenotypes (upper panels). Low-magnification images of the tumor seen in upper panels reveal boundaries between tumor (T) areas and areas of non-tumorous (N) liver parenchyma tissue (middle panels). Images of picro-sirius red staining from the same area seen in middle panels are presented in lower panels. Note the presence of accumulated collagen bands in the area surrounding the liver tumor in an MPC mouse. Scale bars, 200 μm for upper panels and 1 mm for middle and lower panels

To develop HCC in the background of liver fibrosis, we attempted to induce fibrosis in livers expressing cMyc plus shp53 via treatment with carbon tetrachloride (CCl_4_), a hepatotoxic chemical that induces chronic liver damage [15, 16]. Mice were hydrodynamically injected with transposons encoding cMyc plus shp53, and assigned randomly to the CCl_4_ and vehicle-treated groups (*n* = 10 for each group). Treatment was started at 15 d PHI and performed twice per week throughout the experiment (Fig. 2a). Cells transfected with cMyc and shp53 remained as single cells at 15 d PHI and no lesions were observed in the livers (Additional file 1). As a control (referred to as ‘CT’), 10 mice were treated with CCl_4_ without hydrodynamic injection.

A few mice treated with CCl_4_ were severely ill and died within 1 month of treatment. A similar frequency of lethality followed treatment with CCl_4_, regardless of hydrodynamic injection (40 % for mice without hydrodynamic injection vs. 30 % for mice with hydrodynamic injection), indicating that hydrodynamic injection does not affect lethality. The mice treated with the vehicle following hydrodynamic injection did not die. No tumors were detected in the livers of mice that died during this period. None of the mice that survived the first 30 d of CCl_4_ treatment died during the remainder of the study period.

A few mice hydrodynamically transfected with cMyc plus shp53, and treated with CCl_4_ (referred to as “MPC mice”) showed signs of discomfort starting at about 14 weeks after hydrodynamic injection. Livers were harvested from mice of all groups at 102 d PHI, after 87 d of treatment (Fig. 2a). Gross examination revealed that the CT mice (*n* = 6) had no hyperplastic nodules in the liver, although the surface was somewhat rough (Fig. 2b). Two of the ten mice transfected with cMyc plus shp53, and then treated with the vehicle (referred to as “MP mice”), had a single liver tumor. All remaining MPC mice (*n* = 7) had multiple large tumors in the liver (Fig. 2b). Of note, livers of MPC mice had a roughened appearance as seen in those of CT mice.

### Successful generation of a transgenic mouse model of HCC with concurrent hepatic fibrosis

Histopathological examination revealed that liver tumors from both MP and MPC had similarly well-differentiated HCCs (Fig. 2c) [19]. No microscopic nodules were observed in livers from the CT mice, while an increased number of inflammatory cells were observed in the tissue, likely due to liver injury induced by CCl_4_ (Fig. 2c).

**Fig. 3:**
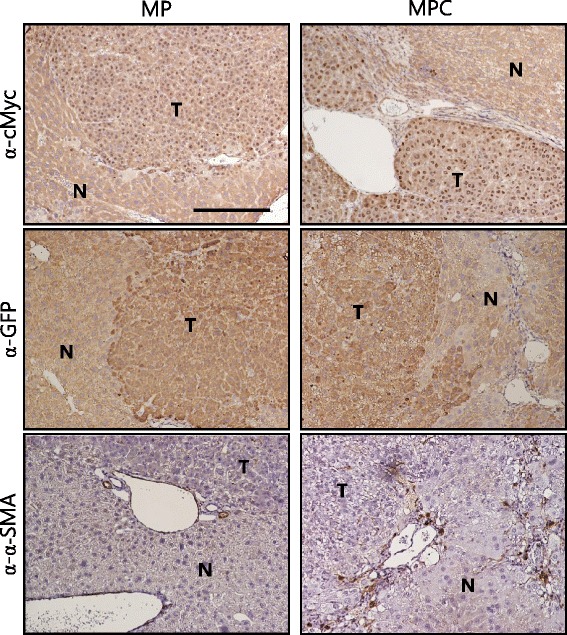
Images of IHC staining for cMyc, GFP and α-SMA in tumor sections. Tumors from both MP and MPC mice were stained positive for cMyc and GFP. Note that cMyc was localized in the nuclei of tumor cells. The area surrounding tumor of MPC mice shows the presence of activated hepatic stellate cells, based on α-SMA staining. “T” denotes tumor and “N” denotes non-tumorous liver parenchyma tissue. Scale bar, 200 μm

Picro-sirius red staining revealed that fibrosis was present throughout the tissues of CT mice (Fig. 2c). Fibrosis was not observed in tumor-bearing livers of MP mice (Fig. 2c). In stark contrast to MP mice, MPC mice had fibrosis in non-tumorous liver parenchyma tissue surrounding the tumors (Fig. 2c). Liver tumors in both MP and MPC mice were GFP and cMyc-positive, indicating that the tumors were induced by transfection with the oncogene-encoding transposons (Fig. 3).

Activation of hepatic stellate cells (HSCs) represents a critical event in fibrosis, mediating secretion of fibrillar collagens and accumulation of the extracellular matrix components [7, 8]. In fibrotic areas around tumors in MPC mice, activated hepatic stellate cells were observed based on immunohistochemistry (IHC) analysis with antibodies against α-SMA, a marker of activated HSC (Fig. 3) [26]. Thus, treatment with CCl_4_ resulted in successful production of a transgenic mouse model for HCC, accompanied by liver fibrosis within less than 4 months at a 100 % incidence.

### Survival of MP and MPC mice

To perform a statistical comparison of the survival of MP and MPC mice, we replicated the experiment using an increased number of mice. MP mice (*n* = 21) and MPC mice (*n* = 31) were monitored for survival following HT. As control groups, mice hydrodynamically transfected with transposons encoding cMyc and GFP were used (Additional file 2). As in the previous experiment, treatment with CCl_4_ or the vehicle was started at 15 d PHI and administered twice per week thereafter.

**Fig. 4:**
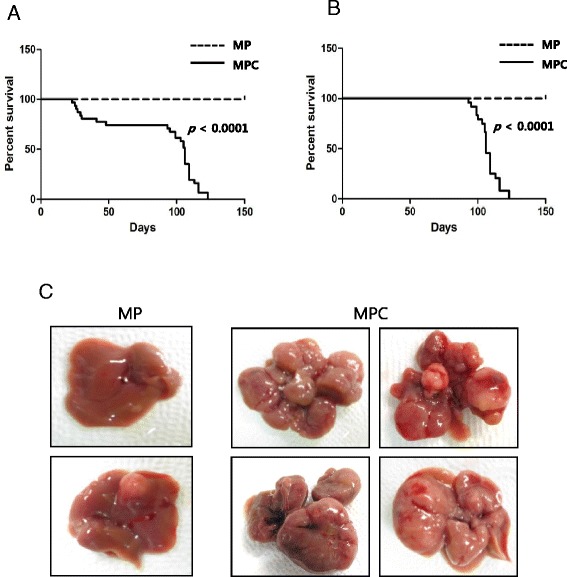
Short life span of MPC mice and increased tumor burdens in their livers. **a**, **b** Kaplan–Meier survival curves of MP and MPC mice in the entire cohort (**a**) and with the exclusion of mice that died initially due to CCl_4_-induced toxicity (**b**). Note that MPC mice had a significantly shorter life span compared to MP mice, even with the exclusion of the mice that died early in the MPC group (*p* < 0.0001). **c** Gross images of representative livers of MP and MPC mice. Livers were harvested following death for the MPC group and at the end of the experiment in the MP group

About 23 % of the mice died within the first 30 d of CCl_4_ treatment in the MPC group (i.e., within 45 days post PHI), as observed in the previous experiment. Again, no tumors were observed in livers of MPC mice that died during the early period. The remaining MPC mice (*n* = 24) showed signs of discomfort, starting around 90 d PHI, and they died between 97 and 125 d PHI. None of the MP mice died or developed illness during this period. The treatment part of the experiment was terminated at 150 d PHI and livers from all MP mice (*n* = 21) were harvested. The Kaplan-Meier survival analysis showed a significantly shorter life span of MPC mice compared to the MP mice (Fig. 4a). Even when mice that had died before 50 d PHI were excluded from the MPC group due to CCl_4_-induced toxicity, a significant difference was observed in survival of the MPC and MP groups (*p* < 10^−4^; Fig. 4b). Therefore, treatment with fibrosis-inducing CCl_4_ significantly shortened the life span of mice expressing cMyc plus shp53. As observed in MPC mice, control mice expressing cMyc and GFP treated with CCl_4_ displayed a similar percentage of lethality (~30 %) within the first 30 d of the treatment, however no death was observed in the group later on until the experiment end point (Additional file 2). Mice that were transfected with cMyc and GFP followed by the vehicle treatment showed no deaths throughout the experiment. The control mice expressing cMyc and GFP exhibited no tumors in their livers regardless of the treatment with CCl_4_ when livers were investigated at 150 d PHI (data not shown).

### Short life span of MPC mice is correlated with effects of liver tumors

To determine if the short life span of MPC mice was due to the liver tumors in the mice, livers were harvested from MPC mice following death. All of the 24 MPC mice that died between 97 and 125 d PHI had multiple large tumors in their livers (Fig. 4c). Livers were harvested from MP mice (*n* = 21) at 150 d PHI (the experiment end point) and inspected for the presence of tumors. Only 38 % of the MP mice (8 of 21 mice) had one or two small nodules in their livers (Fig. 4c). The incidence of HCC in MPC mice was significantly higher than in MP mice (*p* < 10^−4^; Table 1). Inclusion of seven MPC mice that died initially due to CCl_4_-induced toxicity reduced the incidence to 77 % in that group (24 of 31 mice), but the incidence was still significantly higher compared to that for MP mice (*p* < 0.01).

**Table 1:** Summary of tumor incidence in cMyc + shp53 mice treated with vehicle (MP) vs. cMyc + shp53 mice treated with CCl_4_ (MPC) from the survival study. Livers were harvested from MPC mice following death and from MP mice at the end of the experiment

	Total # of mice^a^	% of mice with liver tumors	Average # of tumors per mouse	Total # of mice^b^	% of mice with liver tumors	Average # of tumors per mouse
MP	21	38 % (8/21)^*^	0.6^†^	21	38 % (8/21)^‡^	0.6^§^
MPC	31	77 % (24/31)^*^	9^†^	24	100 % (24/24)^‡^	11.7^§^

^a^Entire cohort

^b^Mice that died initially due to CCl_4_-induced toxicity were excluded (i.e., those died before 50 days post hydrodynamic transfection)

^*^ *P* < 0.01, Fisher’s exact test. ^†^ *P* < 0.0001, Student’s *t*-test

^‡^ *P* < 0.0001, Fisher’s exact test. ^§^ *P* < 0.0001, Student’s *t*-test

The average tumor number of MPC group livers was about 15-fold that in the MP group (*p* < 10^−4^; Table 1). The difference was larger when the seven MPC mice that died early in the study were excluded, as they did not have tumors.

The increased number of tumor nodules observed in MPC mice suggests that the fibrotic environment induced by CCl_4_ treatment enhanced tumor initiation induced by cMyc overexpression and p53 down-regulation. Furthermore, the liver tumors in the MPC group appeared larger than those in the MP group (Fig. 4c), suggesting that the growth of tumors induced by cMyc plus shp53 was accelerated in fibrotic livers. Overall, the increased tumor burden in the liver of MPC mice likely led to a shortened life span.

## Discussion

In the present study, we developed a transgenic model system in which HCC was induced by oncogenic expression in the background of liver fibrosis, mimicking human hepatocarcinogenesis. Treatment with CCl_4_ not only induced liver fibrosis in non-tumorous parenchyma surrounding HCC but also accelerated the carcinogenic process induced by cMyc plus shp53 in the model, developing HCC within 3 months of treatment. Treatment with CCl_4_ alone, even for 6 months, failed to induce tumors in our experiment (data not shown), suggesting that the CCl_4_ treatment could not induce tumor, but promoted the hepatocarcinogenesis induced by cMyc plus shp53. The simplicity and efficiency in inducing HCC, as well as the resemblance to human hepatocarcinogenesis, suggests the utility of this model system in preclinical studies of HCC [12].

Our data showed that CCl_4_ induced background liver fibrosis and significantly enhanced hepatocarcinogenesis initiated by cMyc overexpression and *p53* suppression. However, the molecular mechanism underlying the increased tumorigenesis remains unclear. As a hepatotoxic chemical inducing chronic liver injury, CCl_4_ has been widely used to induce fibrosis in livers. A persistent injury to the liver leads to hepatocyte death, followed by compensatory regeneration, chronic inflammation, accumulation of extracellular matrix components, and subsequent changes in the tissue microenvironment [7, 26, 27]. A regenerative microenvironment promoting cellular proliferation might enhance tumor initiation in cells expressing cMyc plus shp53 [28]. Upregulation of inflammatory cytokines (e.g., IL-6 and TNF-α) in livers with chronic injury is known to support tumor-promoting microenvironments [29, 30]. Finally, the possibility cannot be ruled out that the treatment caused a genetic alteration directly or via upregulation of reactive oxygen species [15, 31], further enhancing tumor initiation induced by cMyc plus shp53. Ongoing studies are needed to investigate the molecular mechanism underlying the increased hepatocarcinogenesis of fibrotic livers in the model.

Several genetically engineered mouse (GEM) models for HCC have been developed, with alterations in candidate oncogenes or tumor suppressor genes, and significantly contributed to a better understanding of the genetic mechanisms underlying hepatocarcinogenesis [10, 11]. Circumventing procedures requiring excessive time and resources in developing a GEM model, a new methodology has been developed for simple generation of a transgenic HCC model, employing HT coupled with the SB transposon system [13, 14]. Many transgenic HCC models have been developed via HT and successfully applied to liver cancer research. The common problem with both traditional genetic models and HT models for HCC is that they do not consider background liver fibrosis. Considering that human HCC develops mostly in a fibrotic or cirrhotic liver, the lack of fibrosis in the background liver could be a critical limitation in the HCC models. Applying the chronic liver injury model, induced by CCl_4_ treatment, to a transgenic HCC model developed by HT, a transgenic HCC model with background liver fibrosis was efficiently developed within a few months. Considering the simplicity and efficiency, as well as the resemblance to human HCC, the model can be effectively applied to preclinical testing of HCC anticancer therapy and studies of hepatocarcinogenesis in fibrotic livers.

## Conclusions

A transgenic mouse model of hepatocellular carcinoma (HCC) with background liver fibrosis was developed, combining a chronic liver injury model with liver transgenesis via hydrodynamics-based transfection. Liver fibrosis significantly accelerated hepatocarcinogenesis induced by cMyc overexpression and p53 suppression.

## Additional files

Additional file 1:
**Histological analysis of livers harvested at 15 days post hydrodynamic transfection with transposons encoding cMyc and shp53.** Description of Data: H&E and IHC images. (PDF 220 kb) Additional file 2:
**Short life span of MPC mice. Description of Data: Survival graphs.** (PDF 209 kb)

## Abbreviations

α-SMA: α-smooth muscle actin
CCl_4_: carbon tetrachloride
CT: CCl_4_-treated mouse
GEM: genetically engineered mouse
GFP: green fluorescent protein
HCC: hepatocellular carcinoma
H&E: hematoxylin & eosin, HSC, hepatic stellate cell
HT: hydrodynamic transfection
IHC: immunohistochemistry
MP: cMyc plus shp53
MPC: cMyc plus shp53 with CCl_4_ treatment
PHI: post hydrodynamic injection
SB: Sleeping Beauty
shp53: short hairpin RNA down-regulating p53
WT: wild-type
