# Supplementary material for: Development of a transgenic mouse model of hepatocellular carcinoma with a liver fibrosis background
Source: BMC Gastroenterol. 2016 Jan 29;16:13. doi: 10.1186/s12876-016-0423-6 (PMC4731926; doi:10.1186/s12876-016-0423-6)
Supplement: Additional file 1: — Histological analysis of livers harvested at 15 days post hydrodynamic transfection with transposons encoding cMyc and shp53. Description of Data: H&E and IHC images. (PDF 220 kb) [file 12876_2016_423_MOESM1_ESM.pdf]

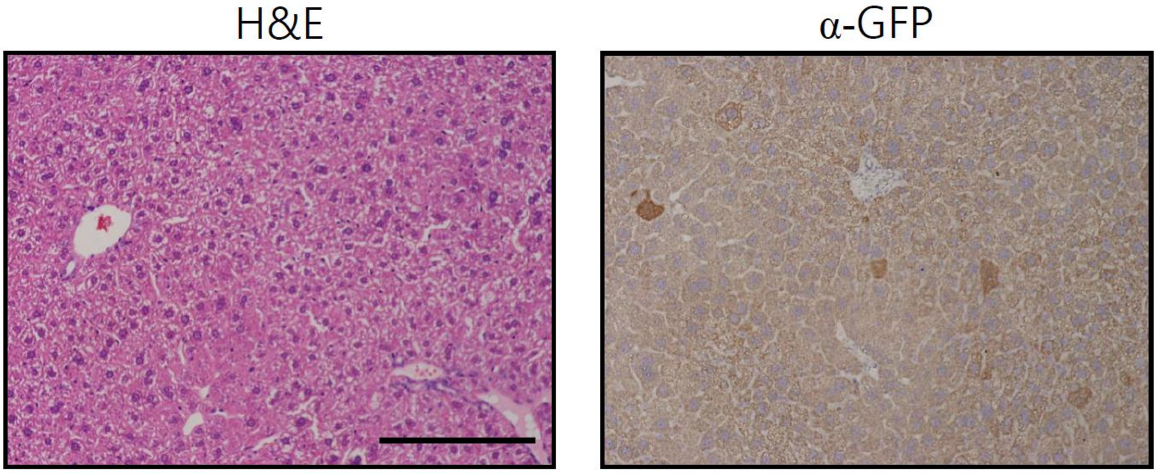

Additional file 1. Histological analysis of livers harvested at 15 days post hydrodynamic transfection with transposons encoding cMyc and shp53. Representative images of H&E and IHC staining for GFP in liver sections. Scale bar, 200 μm.
