# Supplementary material for: Development of a transgenic mouse model of hepatocellular carcinoma with a liver fibrosis background
Source: BMC Gastroenterol. 2016 Jan 29;16:13. doi: 10.1186/s12876-016-0423-6 (PMC4731926; doi:10.1186/s12876-016-0423-6)
Supplement: Additional file 2: — Short life span of MPC mice. Description of Data: Survival graphs. (PDF 209 kb) [file 12876_2016_423_MOESM2_ESM.pdf]

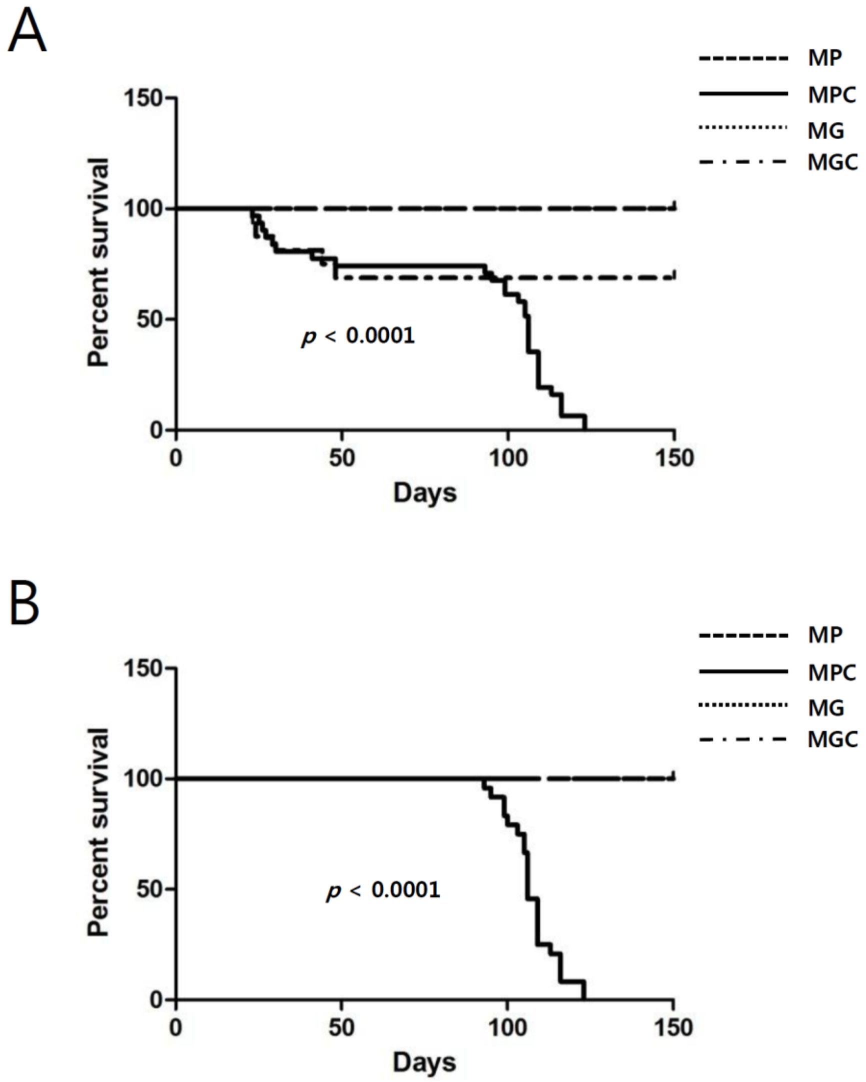

Additional file 2. Short life span of MPC mice. (A), (B) The survival graphs shown in Figure 4 were redrawn with the inclusion of control mice that were transfected with cMyc and GFP and then treated with vehicle (referred to as "MG mice") or CCl_4 (referred to as "MGC mice"). Kaplan–Meier survival curves of MG, MGC, MP, and MPC mice in the entire cohort (A) and with the exclusion of mice that died initially due to CCl_4-induced toxicity (B). Note that MPC mice had a significantly shorter life span compared to mice of the other groups ($p < 0.0001$).
